# Tunable spin-dependent Andreev reflection in a four-terminal Aharonov-Bohm interferometer with coherent indirect coupling and Rashba spin-orbit interaction

**DOI:** 10.1186/1556-276X-7-670

**Published:** 2012-12-10

**Authors:** Long Bai, Rong Zhang, Chen-Long Duan

**Affiliations:** 1 College of Science, China University of Mining and Technology, Xuzhou, 221116, China; 2 School of Chemical Engineering and Technology, China University of Mining and Technology, Xuzhou, 221116, China

**Keywords:** Aharonov-Bohm interferometer, Double quantum dot, Andreev reflection, Rashba spin-orbit interaction, Coherent indirect coupling, 73.63.Kv; 73.23.-b; 72.25.-b

## Abstract

Using the nonequilibrium Green’s function method, we theoretically study the Andreev reflection(AR) in a four-terminal Aharonov-Bohm interferometer containing a coupled double quantum dot with the Rashba spin-orbit interaction (RSOI) and the coherent indirect coupling via two ferromagnetic leads. When two ferromagnetic electrodes are in the parallel configuration, the spin-up conductance is equal to the spin-down conductance due to the absence of the RSOI. However, for the antiparallel alignment, the spin-polarized AR occurs resulting from the crossed AR (CAR) and the RSOI. The effects of the coherent indirect coupling, RSOI, and magnetic flux on the Andreev-reflected tunneling magnetoresistance are analyzed at length. The spin-related current is calculated, and a distinct swap effect emerges. Furthermore, the pure spin current can be generated due to the CAR when two ferromagnets become two half metals. It is found that the strong RSOI and the large indirect coupling are in favor of the CAR and the production of the strong spin current. The properties of the spin-related current are tunable in terms of the external parameters. Our results offer new ways to manipulate the spin-dependent transport.

## Background

A quantum dot (QD) is an artificially low-dimensional structure that can be filled with electrons (or holes). Two or more QDs can be coupled to form multiple-QD systems (i.e., artificial molecules). Because the degrees of freedom of the QDs are well controllable, it is possible to add or remove the electrons in the QDs, and the QD system can be coupled via tunnel barriers to electrodes, in which electrons can be exchanged. Accordingly, the artificial molecule provides an excellent model system in which the thorough investigation of quantum many-body properties in a confined geometry can be performed [1-6]. Among the various multiple-QD systems, an Aharonov-Bohm (AB) interferometer containing double QDs (DQDs) is of particular interest and importance, in which two QDs are embedded in the opposite arms of the AB ring, respectively, and they are coupled to each other via barrier tunneling. As a tunable two-level system, the parallel DQD system that can become one of the promising candidates for the quantum bit in quantum computation has received more attention [7-20]. However, in an actual DQD system, the coherent indirect coupling between two QDs via a reservoir is very essential. Kubo et al. introduced the parameter _1_*α*characterizing the indirect coupling strength, and Gurvitz also indicated the fundamentality of the sign of the coherent indirect coupling parameter [21,22]. Kubo et al. investigated the pseudospin Kondo effect in a lateral DQD system using the slave-boson mean-field method and found that the exotic pseudospin Kondo effect occurs when a coherent indirect coupling is presented through the common reservoirs [23]. Recently, Kubo and co-workers calculated the shot noise and Kondo effect in a DQD structure with the coherent indirect coupling. Their results demonstrate that the coherent indirect coupling can generate a novel antiferromagnetic exchange phenomenon [24]. Trocha and Barnaś studied theoretically the spin-dependent transport through a DQD coupled to ferromagnetic leads. They observed that the Fano antiresonance of the linear conductance relies on the sign of the indirect coupling in the nondiagonal coupling elements [8]. Furthermore, the transport properties of a DQD system has been considered in the orbital Kondo regime. That the Kondo temperature and Kondo resonances are susceptible to the coherent indirect coupling parameter is also revealed [25]. In addition, if a QD is formed in a semiconductor two-dimensional electron gas structure without the inversion symmetry in the growth direction, the Rashba spin-orbit interaction (RSOI) will emerge, and the RSOI can induce the spin-related phase factor in the tunneling matrix elements and the spin-flip effect. The RSOI results from a relativistic effect at the low speed limit, and it can couple the electron spin to its orbital motion, thus providing a possible way to control the spin degree of freedom by means of an external electric field. As a consequence, the coherent indirect coupling and the RSOI make the quantum transport through the QD systems rich and varied [26-30].

On the other side, the subgap transport through heterostructures with nano-objects (such as QDs, molecules, nanowires, etc.) coupled to one conductor and another superconducting lead has attracted a great deal of attention over the past years due to the fundamental physics and its potential applications [31-35]. Andreev reflection (AR) usually occurs in the hybrid systems, in which two electrons with opposite spins enter the superconductor from the normal metal region, leading to the formation of a Cooper pair in the superconducting region [36-38]. In comparison with the standard mechanism of normal AR, the crossed AR (CAR) is a nonlocal dynamics process which occurs at the contact between a superconductor and two normal leads, where two subgap electrons from different metals enter into the superconductor and generate a Cooper pair there [39-42]. AR (or CAR) in nanoscopic heterostructures gives rise to a rich subgap structure in the current-voltage characteristics. Accordingly, understanding the AR and CAR has attracted theoretical and experimental attention mainly because the AR (or CAR) may create the entangled electrons in a solid-state device, and CAR can be readily probed by spin selection using ferromagnetic electrodes. This approach is almost unrealized for entangler devices, since projecting the spin will cause the destruction of entanglement [43]. Based on the CAR, the controlled Cooper pair splitting has been realized in terms of a two-quantum dot Y-junction [44], which opens a possible route towards a test of the Einstein-Podolsky-Rosen (EPR) paradox and Bell inequalities in solid-state systems. Herrmann et al. used carbon nanotube DQD as Cooper pair beam splitters and realized the quantum optic-like experiments with spin-entangled electrons [45]. These results show that the CAR has an important application in testing a fundamental property of quantum mechanics.

To our knowledge, the AR in the DQD with a maximum coupling |*α*| = 1 has been studied widely. However, the quantum transport through a four-terminal AB interferometer including a DQD in the presence of the AR, the coherent indirect coupling, and RSOI is less explored. Motivated by recent theoretical and experimental advances in the DQD systems [7-10,13,15,16,19,21-25,44,45], one may expect that the interplay of the coherent indirect coupling and the RSOI in the presence of the AR will add new physics to hybrid quantum systems, which may have practical applications for future spintronics. Consequently, we investigate the AR in the above-mentioned system in this paper. It is found that the RSOI and a nonzero coherent indirect coupling cause the spin-polarized AR when the polarizations of two ferromagnetic leads are parallel, but for antiparallel (AP) arrangement of the polarizations of two ferromagnetic leads, the CAR can contribute the spin-polarized AR conductance. We note that the convex shape of the Andreev-reflected tunneling magnetoresistance (ARTMR) versus the magnetic flux relies on the sign of the coherent indirect coupling parameter, and there are extreme values in the plot of the ARTMR versus the coherent indirect coupling parameter. Even the negative ARTMR also occurs. This is a spin valve effect in the AR process. It is interesting to note that the sign of the coherent indirect coupling parameter leads to the swap effect in the spin-polarized current plot, and the pure spin current can be produced when two ferromagnetic leads are fully polarized. The spin-dependent AR current can be controlled by means of the gate voltage, RSOI, magnetic flux, and so on. These results provide the ways to manipulate the spin-dependent transport by means of the system parameters.

## Methods

**Figure 1:**
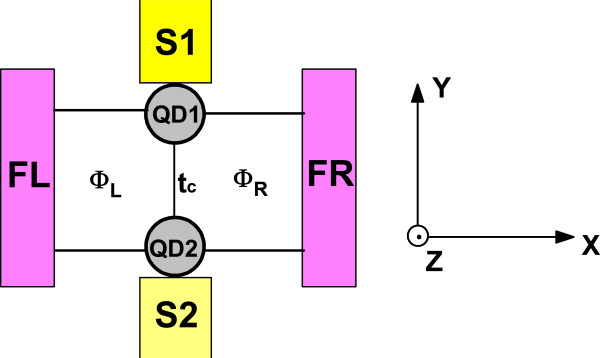
**Schematic diagram of a four-terminal AB interferometer (color on line).** The AB interferometer contains a coupled DQD with magnetic flux applied perpendicular to rings.

We consider a hybrid four-terminal AB interferometer including a parallel DQD coupled to two ferromagnetic reservoirs and two superconductors as shown in Figure 1. The system is described by the following Hamiltonian:

(1) $$H=H_{F}+H_{S}+H_{\text{DQD}}+H_{T},$$

where *H*_F _is the Hamiltonian of the left and right ferromagnetic electrodes

(2) $$H_{F}=\underset{\nu =L,R}{\sum }\underset{k\sigma }{\sum }\varepsilon_{\nu ,k\sigma }c_{\nu ,k\sigma }^{†}c_{\nu ,k\sigma }.$$

Here, $\left(c_{\nu ,k\sigma }^{†}(c_{\nu ,k\sigma })\right)$ is the creation (annihilation) operator in the lead *ν*with energy *ε*_*ν*,*kσ*_. *H*_S_ represents two superconducting reservoirs with chemical potential *μ*_s _= 0 and the energy gap Δ,

(3) $$H_{S}=\underset{\gamma =1,2}{\sum }\underset{k\sigma }{\sum }\varepsilon_{\gamma ,k\sigma }c_{\gamma ,k\sigma }^{†}c_{\gamma ,k\sigma }+\underset{k}{\sum }(\Delta c_{\gamma ,k↑}^{†}c_{\gamma ,-k↓}^{†}+\text{h.c.}).$$

*H*_DQD_ in Equation 1 denotes the DQD Hamiltonian

(4) $$H_{\mathrm{DQD}}=\underset{i\sigma }{\sum }\varepsilon_{i}d_{i\sigma }^{†}d_{i\sigma }+t_{c}\underset{\sigma }{\sum }(d_{1\sigma }^{†}d_{2\sigma }+\text{h.c.}),$$

in which $\left(d_{i\sigma }^{†}(d_{i\sigma },i=1,2)\right)$ represents the creation (annihilation) operator of the electron with energy *ε*_*i*_ in the dot *i*; *t*_c_ is the coupling strength taken as a real parameter. The last term, *H*_T_, in Equation 1 corresponds to the tunneling Hamiltonian between the DQD and four leads,

(5) $$H_{T}=\underset{\text{ki}\nu \sigma }{\sum }(T_{\nu ,k}^{\left(i\right)}c_{\nu ,k\sigma }^{†}d_{i\sigma }+\text{h.c.})+\underset{\text{ki}\nu \sigma }{\sum }(T_{\gamma ,k}^{\left(i\right)}c_{\gamma ,k\sigma }^{†}d_{i\sigma }+\text{h.c.}),$$

where the tunneling matrix elements between the DQD and two ferromagnetic leads are $\left(T_{L,k}^{\left(1\right)}=|T_{L1}|e^{i\phi /4}\right)$, $\left(T_{L,k}^{\left(2\right)}=|T_{L2}|e^{-i\phi /4}\right)$, $\left(T_{R,k}^{\left(1\right)}=|T_{R1}|e^{-i\phi /4}e^{-i\varphi_{R1}/4}\right)$, and $\left(T_{R,k}^{\left(2\right)}=|T_{R2}|e^{i\phi /4}e^{i\varphi_{R2}/4}\right)$. The phase shift due to the total magnetic flux threading into the AB ring is assumed to be *ϕ *= 2*Π*(Φ_L_ + Φ_R_)/*ϕ*_0_ with the flux quantum *ϕ*_0 _=* h*/*e*. The phase factor *φ*_*Ri *_comes from the RSOI in dot *i*, which is tunable in the experiments [46,47]. $\left(T_{\gamma ,k}^{\left(i\right)}=T_{S1}(T_{S2})\right)$ as the tunneling coupling between the DQD and two superconductors is also assumed to be independent of *k* and *σ*.

Using the nonequilibrium Green’s function technique, the spin-dependent current through the left ferromagnetic reservoir can be expressed as [48,49]

(6) $$I_{L\sigma }=\frac{\text{ie}}{\hbar }\int \frac{d\varepsilon }{2\Pi }\mathrm{Tr}({\hat{\sigma }}_{z}\Gamma_{L}\{G^{<}(\varepsilon )+F_{L}[G^{r}(\varepsilon )-G^{a}(\varepsilon )]\}),$$

where Tr is the trace in the spin space; $\left({\hat{\sigma }}_{z}\right)$ is a 4 × 4 matrix with Pauli matrix *σ*_*z*_ as its diagonal components; *G*^r,*a*,<^(*ε*) are retarded, advanced, and lesser Green’s functions in the generalized 4 × 4 Nambu notation.

(7) $$G^{r}(t,t^{\prime })=-i\theta (t-t^{\prime })\langle \{\Psi (t),\Psi^{†}(t^{\prime })\}\rangle ,$$

(8) $$G^{<}(t,t^{\prime })=i\langle \Psi^{†}(t^{\prime })\Psi (t)\rangle ,$$

with the vector $\left(\Psi^{†}=(d_{1↑}^{†},d_{1↓},d_{2↑}^{†},d_{2↓})\right)$.

After some algebraic manipulations, the spin-dependent current can be derived from Equation 6:

(9) $$\begin{array}{l} I_{L↑}=\frac{e}{h}\int d\varepsilon [T_{↑}^{\text{AR}}(\varepsilon )(f_{L}-{\bar{f}}_{L})+T_{↑}^{\text{CAR}}(\varepsilon )(f_{L}-{\bar{f}}_{R})\\ \;+T_{↑}^{\mathrm{LR}}(\varepsilon )(f_{L}-f_{R})+T_{↑}^{\mathrm{QS}}(\varepsilon )(f_{L}-f_{S})], \end{array}$$

(10) $$\begin{array}{l} I_{L↓}=\frac{e}{h}\int d\varepsilon [T_{↓}^{\text{AR}}(\varepsilon )(f_{L}-{\bar{f}}_{L})+T_{↓}^{\text{CAR}}(\varepsilon )(f_{R}-{\bar{f}}_{L})\\ \;+T_{↓}^{\mathrm{LR}}(\varepsilon )({\bar{f}}_{R}-{\bar{f}}_{L})+T_{↓}^{\mathrm{QS}}(\varepsilon )(f_{S}-{\bar{f}}_{L})], \end{array}$$

in which $\left(T_{\sigma }^{\text{AR}}\right)$ and $\left(T_{\sigma }^{\text{CAR}}\right)$ are the spin-dependent AR and CAR coefficients, respectively. $\left(T_{\sigma }^{\mathrm{LR}}\right)$ represents the single-particle tunneling through FL-DQD-FR or FR-DQD-FL. $\left(T_{\sigma }^{\mathrm{QS}}\right)$ corresponds to the probability of the quasiparticle tunneling among two superconductors and the left ferromagnetic lead. $\left(f_{L}({\bar{f}}_{L})\right)$, $\left(f_{R}({\bar{f}}_{R})\right)$, and *f*_S_ are Fermi-Dirac distribution functions. The derivation of the spin-dependent current is minutely given in the Appendix.

Since we mainly focus on the AR process at zero temperature limit and set |*eV*_L_| = |*eV*_R_| < Δ, $\left(T_{\sigma }^{\mathrm{QS}}\right)$ will vanish. In the case of *eV*_L _=* eV*_R_, the current from the quasiparticle tunneling through FL-DQD-FR or FR-DQD-FL becomes zero; as a consequence, the AR dominates the transport through the four-terminal AB interferometer.

## Results and discussions

In the following numerical calculations, we mainly elucidate the spin-dependent AR process in the four-terminal AB interferometer with the coherent indirect coupling and the RSOI. We take *e *=* h *=* k*_*B *_= 1, and set Δ = 1 as the energy unit. Throughout the paper, the symmetric couplings with $\left(\Gamma_{1}^{\nu }=\Gamma_{2}^{\nu }=\Gamma_{s}=\Gamma =0.2\Delta \right)$ and |*P*_L_| = |*P*_R_| are considered as a typical case.

### Conductance

Because we mostly investigate the AR within the superconductor gap, in the limit of zero bias *V*_L_ =* V*_R _→ 0, the spin-related AR and CAR conductances have the forms

(11) $$G_{\sigma }^{\text{AR}}=\frac{2e^{2}}{h}T_{\sigma }^{\text{AR}}(\varepsilon_{F})$$

and

(12) $$G_{\sigma }^{\text{CAR}}=\frac{2e^{2}}{h}T_{\sigma }^{\mathrm{CAR}}(\varepsilon_{F}).$$

It is well known that a DQD system with the maximum coupling |*α*| = 1 has already been investigated. Indeed, such case is very special, and most experimental conditions correspond to |*α*| < 1; as a result, *α *characterizing the coherent indirect coupling between two QDs via two ferromagnetic electrodes is introduced (see the Appendix). |*α*| < 1 comes from the various factors, such as imperfections in the ferromagnetic reservoirs producing the destructive quantum interference, the geometrical structure of the system, and so forth.

**Figure 2:**
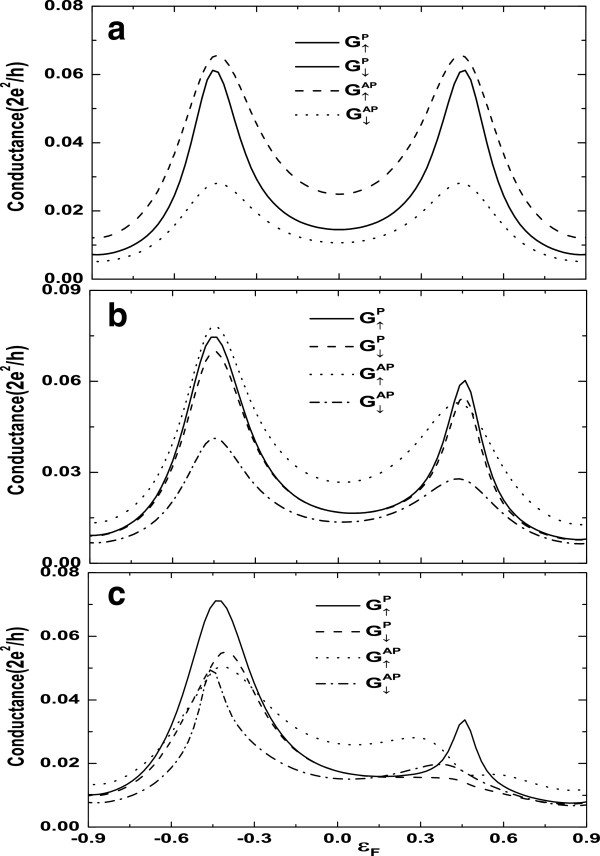
**The AR conductance versus Fermi energy for P and AP configurations.** (**a**) *α *= 0, (**b**) *α *= 0*.*5, and (**c**) *α *= 1*.*0. Other parameters are *ε*_1 _=* ε*_2 _= 0, *t*_c _= 0*.*5, *ϕ *= 0, and *φ*_R _=* Π*/2.

Let us begin with the case of *ϕ *= 0 and *φ*_R _=* Π*/2; for the different coherent indirect coupling *α*, Figure 2 shows the total AR conductance ( $\left(G_{\sigma }^{P}=G_{\sigma }^{\text{AR}(P)}+G_{\sigma }^{\text{CAR}(P)}\right)$ and $\left(G_{\sigma }^{\text{AP}}=G_{\sigma }^{\text{AR}(\text{AP})}+G_{\sigma }^{\text{CAR}(\text{AP})}\right)$) as a function of Fermi energy *ε*_*F *_for parallel (P) and antiparallel (AP) configurations. In order to gain the clear physics, the Hamiltonian *H*_DQD_ is diagonalized, and two energy eigenvalues are given as $\left(E_{\pm }=\frac{1}{2}[(\varepsilon_{1}+\varepsilon_{2})\pm \sqrt{{\left(\varepsilon_{1}-\varepsilon_{2}\right)}^{2}+4t_{c}^{2}}]\right)$; thus, when the Fermi level coincides with the *E*_+ _and *E*_−_, the resonant AR occurs and two peaks of AR conductances are located around the level *E*_±_ as illustrated in Figure 2a, b, c. For *α *= 0, it is clearly seen that $\left(G_{↑}^{P}\right)$ is always equal to $\left(G_{↓}^{P}\right)$ in the P arrangement (*P*_L _=* P*_R _= 0*.*4), and the magnitudes of two peaks are equal. However, for the case of the AP configuration (*P*_ L_= −*P*_R _= 0*.*4), $\left(G_{↑}^{\mathrm{AP}}\neq G_{↓}^{\mathrm{AP}}\right)$ appears when *α *= 0. Because the ferromagnetic leads have majority and minority electrons, the AR and the CAR are governed by the minority electrons for P configuration; thus, the AR and the CAR do not contribute the spin-polarized transport. For AP alignment, although the AR cannot produce the spin-polarized current, $\left(G_{↑}^{\mathrm{AP}}\neq G_{↓}^{\mathrm{AP}}\right)$ emerges due to the CAR process, in which the CAR is governed by the majority electrons. This leads to the appearance of the spin-polarized conductance. Since two dots are indirectly coupled via two ferromagnetic leads, which is reflected in the nondiagonal coupled matrix elements (see Equations 15 and 16), *α *= 0 implies that the off-diagonal matrix elements vanish due to complete destructive quantum interference; thus, two dots are totally decoupled through two ferromagnetic leads. The AR (or the CAR ) can happen only through QD1 and QD2, respectively. This leads to the conductance $\left(G_{↑}^{P}=G_{↓}^{P}\right)$ for the P arrangement and the equal height of two peaks ( $\left(G_{↑}^{\mathrm{AP}}\right)$ or $\left(G_{↓}^{\mathrm{AP}}\right)$) for the AP configuration.

We also notice that both $\left(G_{↑}^{P}\neq G_{↓}^{P}\right)$ and $\left(G_{↑}^{\mathrm{AP}}\neq G_{↓}^{\mathrm{AP}}\right)$ occur with the increase of *α* for P and AP configurations; thus, $\left(G_{↑}^{P}-G_{↑}^{P}\right)$ is nonzero at *α *≠ 0, which means the occurrence of the spin-polarized AR for P configuration in the presence of the RSOI and the nonzero parameter *α*. As a matter of fact, we have found that $\left(G_{↑}^{P}\equiv G_{↓}^{P}\right)$ is independent of the parameter *α *for P configuration in the absence of the RSOI, which is not shown here. In comparison with the case of *α *= 0, the symmetry of AR conductances with respect to the Fermi energy is significantly broken when *α *≠ 0. It is noticeable that amplitudes of conductance peaks near the level *E*_+ _decrease, and the magnitude of the right peaks is smaller than that of the left ones. In addition, the positions of peaks for AP alignment are also shifted with *α *= 1. These results indicate that the coherent indirect coupling and the RSOI play an important role in determining the feature of the AR conductance spectra.

To elucidate better the properties of the AR under P and AP configurations, in analogy with the conventional tunneling magnetoresistance (TMR) effect of ferromagnetic tunnel junctions, the ARTMR is introduced and defined as

(13) $$\text{ARTMR}=\frac{\left[(G_{↑}^{\mathrm{AP}}+G_{↓}^{\mathrm{AP}})-(G_{↑}^{P}+G_{↓}^{P})\right]}{\left(G_{↑}^{\mathrm{AP}}+G_{↓}^{\mathrm{AP}}\right)}.$$

**Figure 3:**
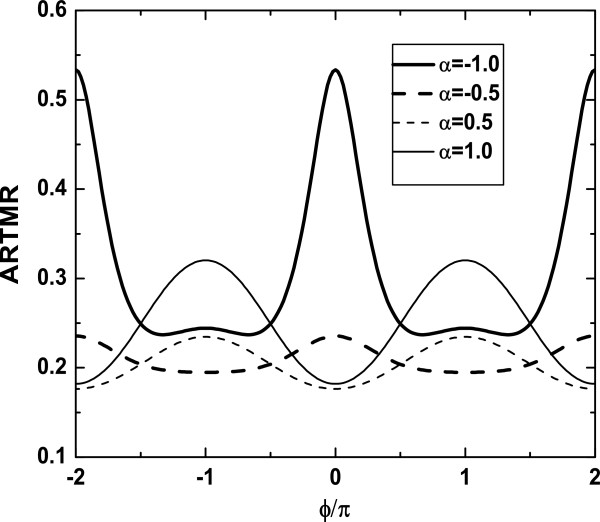
**ARTMR versus the magnetic flux *ϕ *with different *α*.** Other parameters are ε_1 _= ε_2 _= 0, t_c _= 0.5, and *φ*_R _= Π/2

In Figure 3, we present the *ϕ* dependence of ARTMR for different *α*. The oscillation period of the ARTMR versus magnetic flux *ϕ *is 2*Π*, and the sign of the ARTMR does not change. It is interesting to note that the convex shape of the ARTMR at *ϕ *= 2*nΠ *(*n* is an integer) relies on the sign of the coherent indirect coupling parameter *α*. In comparison to the case of |*α*| = 0*.*5, the magnitudes of ARTMR are considerably increased for |*α*| = 1*.*0. This is because the reduction of the destructive interference results in the enhancement of ARTMR.

**Figure 4:**
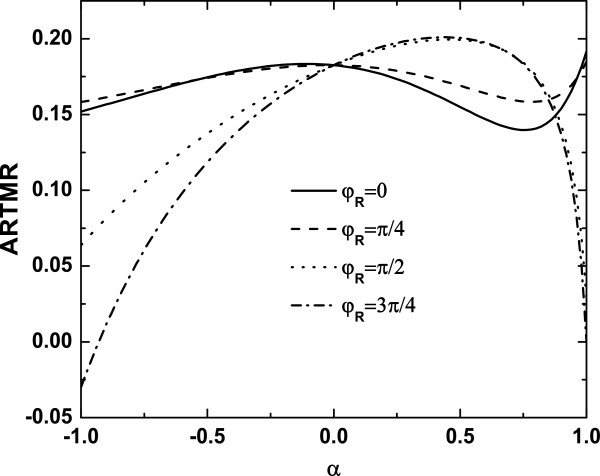
**ARTMR versus the coherent indirect coupling *α *with different *φ***_**R**_**.** Other parameters are *ε*_1 _=* ε*_2 _= 0, *t*_c _= 0.5, and *ϕ* = 0.

As we know, the RSOI can induce the spin precession and may even cause the inter-dot spin-flip effect. According to [26,27], the spin-dependent phase factor *φ*_R_ due to the RSOI can be expressed as $\left(\varphi_{R}=\varphi_{R1}-\varphi_{R2}=\beta m^{*}(L_{1}-L_{2})/\hbar^{2}\right)$, where *β* is the RSOI strength, *m*^∗^ is the electron effective mass, and *L*_*i*_ is the length of dot i. *φ*_R_ is tunable in experiments. It can reach *Π*/2 easily or can be larger experimentally [27]. In order to explore further the influence of the coherent indirect coupling and the RSOI on the ARTMR, the ARTMR as a function of the parameter *α* for different *φ*_R _is shown in Figure 4. We can see from Figure 4 that ARTMR versus *α* exhibits the nonmonotonic features, and there exists the crossing point at *α* = 0. Since *α* = 0 means that the coupling off-diagonal terms in Equation 16 are totally suppressed, as a consequence, the AMTMR is independent of the RSOI (see the Appendix). When *φ*_R _is relatively small, this corresponds to the weak RSOI strength; thus, the variation of the ARTMR with *α* is not smart (solid line and dashed line). However, the evolution of the ARTMR is very remarkable as *φ*_R _increases (dotted line and dash-dotted line), while the ARTMR first increases and decreases with the increases of *α*, even the negative ARTMR also emerges, which corresponds to a spin valve effect in the AR process. This reflects that the strong RSOI gives rise to the significant variation of the ARTMR. We also observe that the maximum and minimum values appear in the curves of the ARTMR. These demonstrate that the optimal ARTMR can be tuned by means of the external parameters.

### Spin-dependent current

**Figure 5:**
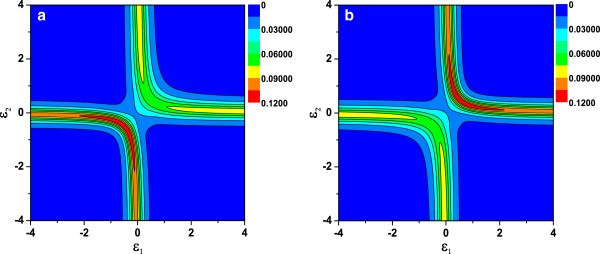
**Images of spin-polarized AR current current as a function of QD levels *ε***_**1 **_**and *ε***_**2 **_**(Color on line).** (**a**)* α *= −0.5 and (**b**) *α *= 0.5. Other parameters are *ϕ*=0, *φ*_*R *_= 0, *t*_c _= 0.5, and *P*_*L *_=* P*_*R *_= 0.4.

Above, we analyze the properties of the AR conductances. In the following discussions, we will explore the spin-dependent current in the AR process with the help of the current formulas (Equations 9 and 10). To gain a full physical picture on the DQD levels’ influence on the spin-related current, Figure 5 displays the images of the spin-polarized current *I*_s _= *I*_L↑ _− *I*_L↓_ as a function of the energy levels *ε*_1_ and *ε*_2_ of the DQD. The blue regions correspond to zero current, namely, *I*_L↑ _= *I*_L↓ _in these regimes. In the diagram, it is found that the spin-polarized current is symmetrical about the line of *ε*_1 _= *ε*_2_ and is asymmetrical with respect to the line of *ε*_1 _= −*ε*_2 _as illustrated in Figure 5a, b. It is interesting to note that one level is aligned to the Fermi level, and the other is far from the Fermi one (off-resonance). I_s _is relative small. This is because one QD is in the on-resonance state and the other is in the off-resonance state. When both *ε*_1_ and *ε*_2_ are close to the Fermi level by tuning the gate voltage, the maximal I_s_appears since DQD is in the on-resonance states. We also observe that, for *α* = 0.5 and *α* = −0.5, the spin-polarized current shows the opposite feature, which is a swap effect originating from the different sign of the parameter *α*. This indicates that the sign of the coherent indirect coupling parameter has a remarkable impact on the spin-polarized current.

**Figure 6:**
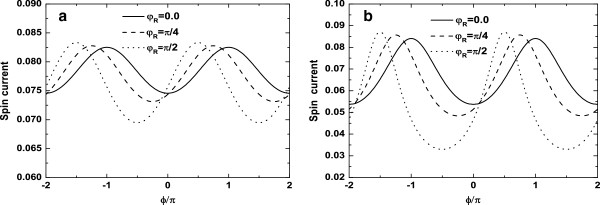
**The spin current versus the magnetic flux *ϕ *for different *φ***_**R **_**.** (**a**)* α *= 0.5 and (**b**) *α *= 1.0. Other parameters are *ε*_1 _=* ε*_2 _= 0, *t*_c _= 0*.*5, *P*_L _= −*P*_R _= 1*.*0

As we know, when ferromagnets are fully polarized, two ferromagnets become half metals where all electrons have the same spin. AR is usually suppressed at the ferromagnet/superconductor interface. However, AR still can occur, and the pure spin current can be generated in the present system. For *P*_L _= −*P*_R _= 1.0 or *P*_L _= −*P*_R _= −1.0, i.e., two ferromagnetic leads become two half metals; the normal AR vanishes due to $\left(T_{\sigma }^{\text{AR}}\right)$ (see Equations 21 and 25). However, CAR dominates the transport through the four-terminal AB interferometer for AP alignment. As a consequence, we can obtain the pure spin current via CAR and two half-metal reservoirs. Thus, this device may be used as a pure spin-current injector even in the absence of the RSOI. In Figure 6, we also depict AB oscillations of the spin current for different *α*. For the case of *α* = 0.5, the magnitudes of the resonant peaks and valleys are enhanced with the increase of the RSOI strength, and positions of peaks and valleys are also shifted to the left, as illustrated in Figure 6a, b. Since the RSOI gives rise to an extra spin-related phase factor *φ*_R _(see Equation 16), the curves of the spin current versus magnetic flux ϕ move towards the left with the emergence of the RSOI phase, and the shifted magnitude of peaks (or valleys) is equal to *φ*_R_ as shown in Figure 6a, b. Physically, the increase of *φ*_R _corresponds to the strong RSOI, which also favors the CAR process and the generation of the large spin current. When the DQD is fully coupled via two ferromagnetic reservoirs (*α *= 1.0), in comparison with the case of *α* = 0.5, it is noted from Figure 6b that not only the positions of peaks and valleys are altered, but also the amplitudes of those are remarkably enhanced. This originates from the fact that the reduction of the destructive interference enhances the spin current for the case of *α* = 1.0. These results indicate that the variation of the spin current is sensitive to the parameter *α* and the strength of the RSOI, and the interplay between them determines the nature of the spin current.

## Conclusions

In this paper, we have analyzed the AR of a four-terminal AB interferometer containing a coupled DQD with with the RSOI and the coherent indirect coupling via two ferromagnetic leads. The formulas of the transmission coefficients are derived based on the framework of the nonequilibrium Green’s function technique. For P configuration, the spin-polarized AR can occur, stemming from the RSOI and a nonzero coherent indirect coupling. On the contrary, for AP configuration, the spin-polarized AR always happens because of the CAR mechanism. Under the introduction of the ARTMR, we find that the sign of the ARTMR versus the magnetic flux keeps invariable for different parameter *α*, but the convex shape of the ARTMR depends distinctly on the sign of the parameter *α*. With the increase of the RSOI strength, the ARTMR versus the parameter *α* exhibits the more significant nonmonotonic features, and there exist the extreme values in the ARTMR plot, even the negative ARTMR also emerges. Since the energy levels of the DQD can be manipulated via the gate voltage, we can obtain the optimal spin-polarized current. A pure spin current can be generated via the CAR and two half-metal leads. Moreover, the strong RSOI and the reduction of the destructive interference (*α *= 1) favor the enhancement of the spin current. Thus, this device may become an effective spin-current generator, and the pure spin current is tuned in terms of the magnetic flux, the RSOI strength, and so forth. These results offer the ways to manipulate the spin-dependent transport via the four-terminal AB setup.

## Appendix

In this appendix, we present the derivation of the current formulas in detail.

Let *g*^r^(*ε*) and *G*^r^(*ε*) denote the retarded Green’s function of the DQD without and with the coupling to the external reservoirs. In the Nambu space, *g*^r^(*ε*) can be given as

(14) $${\left[g^{r}(\varepsilon )\right]}^{-1}=\left(\begin{array}{cccc} \varepsilon -\varepsilon_{1} & 0 & -t_{c} & 0\\ 0 & \varepsilon +\varepsilon_{1} & 0 & t_{c}\\ -t_{c} & 0 & \varepsilon -\varepsilon_{2} & 0\\ 0 & t_{c} & 0 & \varepsilon +\varepsilon_{2} \end{array}\right).$$

Based on the following Dyson equation, the retarded Green’s function of the system can be written as *G*^r^(*ε*)]^−1 ^=* g*^r^(*ε*)^−1^−*Σ*^r^, in which $\left(\Sigma^{r}=\Sigma_{L}^{r}+\Sigma_{R}^{r}+\Sigma_{S1}^{r}+\Sigma_{S2}^{r}\right)$. The lesser Green’s function *G*^<^(*ε*) =* G*^r^(*ε*)*Σ*^<^*G*^a^(*ε*), where *G*^a^(*ε*) =* G*^r^(*ε*)]^*† *^and $\left(\Sigma^{<}=\Sigma_{L}^{<}+\Sigma_{R}^{<}+\Sigma_{S1}^{<}+\Sigma_{S2}^{<}\right)$ In the wide-band limit approximation, the retarded self-energy can be derived from the definition

(15) $$\begin{array}{l} \Sigma_{L}^{r}=-\frac{i}{2}\Gamma_{L}\\ \;=-\frac{i}{2}\left(\begin{array}{cccc} \Gamma_{1↑}^{L} & 0 & \alpha \sqrt{\Gamma_{1↑}^{L}\Gamma_{2↑}^{L}}e^{i\phi /2} & 0\\ 0 & \Gamma_{1↓}^{L} & 0 & \alpha \sqrt{\Gamma_{1↓}^{L}\Gamma_{2↓}^{L}}e^{-i\phi /2}\\ \alpha \sqrt{\Gamma_{1↑}^{L}\Gamma_{2↑}^{L}}e^{-i\phi /2} & 0 & \Gamma_{2↑}^{L} & 0\\ 0 & \alpha \sqrt{\Gamma_{1↓}^{L}\Gamma_{2↓}^{L}}e^{i\phi /2} & 0 & \Gamma_{2↓}^{L} \end{array}\right), \end{array}$$

(16) $$\begin{array}{l} \Sigma_{R}^{r}=-\frac{i}{2}\Gamma_{R}\\ \;=-\frac{i}{2}\left(\begin{array}{cccc} \Gamma_{1↑}^{R} & 0 & \alpha \sqrt{\Gamma_{1↑}^{R}\Gamma_{2↑}^{R}}e^{-i\phi_{\sigma }/2} & 0\\ 0 & \Gamma_{1↓}^{R} & 0 & \alpha \sqrt{\Gamma_{1↓}^{R}\Gamma_{2↓}^{R}}e^{i\phi_{\sigma }/2}\\ \alpha \sqrt{\Gamma_{1↑}^{R}\Gamma_{2↑}^{R}}e^{i\phi_{\sigma }/2} & 0 & \Gamma_{2↑}^{R} & 0\\ 0 & \alpha \sqrt{\Gamma_{1↓}^{R}\Gamma_{2↓}^{R}}e^{-i\phi_{\sigma }/2} & 0 & \Gamma_{2↓}^{R} \end{array}\right), \end{array}$$

(17) $$\begin{array}{l} \Sigma_{S1}^{r}=-\frac{i}{2}\Gamma_{S1}=-\frac{i}{2}\Gamma_{1}\rho_{1}(\varepsilon )\left(\begin{array}{cccc} 1 & -\frac{\Delta }{\varepsilon } & 0 & 0\\ -\frac{\Delta }{\varepsilon } & 1 & 0 & 0\\ 0 & 0 & 0 & 0\\ 0 & 0 & 0 & 0 \end{array}\right), \end{array}$$

(18) $$\begin{array}{l} \Sigma_{S2}^{r}=-\frac{i}{2}\Gamma_{S2}=-\frac{i}{2}\Gamma_{2}\rho_{2}(\varepsilon )\left(\begin{array}{cccc} 0 & 0 & 0 & 0\\ 0 & 0 & 0 & 0\\ 0 & 0 & 1 & -\frac{\Delta }{\varepsilon }\\ 0 & 0 & -\frac{\Delta }{\varepsilon } & 1 \end{array}\right), \end{array}$$

where $\left(\Gamma_{\text{ij}\sigma }^{\nu }=2\Pi \underset{k\sigma }{\sum }T_{\nu ,k}^{(i)*}T_{\nu ,k}^{\left(j\right)}\rho_{\nu \sigma }\right)$ with *ρ*_*νσ*_ being the density of states of the spin *σ *band in the lead *ν*. We calculate the tunneling matrix element by means of the Bardeen’s formula, i.e., $\left(T_{\nu ,k}^{\left(i\right)}=\hbar^{2}\int_{S}[\psi_{\nu ,k}(\vec{r})\nabla \psi_{d}^{(i)*}(\vec{r})-\psi_{d}^{(i)*}(\vec{r})\nabla \psi_{\nu ,k}(\vec{r})]\text{dS}/2m_{e}\right)$, where *m*_e_ is the effective mass, S is the region of the integration, $\psi_{\nu ,k}(\vec{r})$ is the wave function of evanescent mode of the lead *ν*, and $\left(\psi_{d}^{\left(i\right)}\right)$ is the wave function of an electron localized in the QD *i*. Considering the propagation of electrons in the reservoir *ν*, this propagation process (the wave number dependence of $\left(T_{\nu ,k}^{\left(i\right)}\right)$) induces the coherent indirect coupling via the reservoir *ν *between two QDs, which is characterized with the parameter *α*^*ν*^. We assume that (*X*_*i*_, *Y*_*i*_, 0) is the center position of the *i*th QD, *X*_1 _=* X*_2 _=* X*_*D *_and *L *= |*Y*_1_−*Y*_2_|. thus, *α*^*ν*^ is given by $\left(\alpha^{\nu }=\alpha ∼{\left(2X_{D}\right)}^{3}/{\left[L^{2}+{\left(2X_{D}\right)}^{2}\right]}^{\frac{3}{2}}\right)$ based on [21]. We find |*α*| ≤ 1 and decreases with *L*, and |*α*| = 1 corresponds to *L *= 0. We define $\left(\alpha \equiv \Gamma_{12\sigma }^{\nu }/\sqrt{\Gamma_{11\sigma }^{\nu }\Gamma_{22\sigma }^{\nu }}\right)$, in which $\left(\Gamma_{11\sigma }^{\nu }=\Gamma_{1\sigma }^{\nu }\right)$, $\left(\Gamma_{22\sigma }^{\nu }=\Gamma_{2\sigma }^{\nu }\right)$. With the definition of the spin polarization $\left(P_{\nu }=(\Gamma_{i↑}^{\nu }-\Gamma_{i↓}^{\nu })/(\Gamma_{i↑}^{\nu }+\Gamma_{i↓}^{\nu })\right)$ in the lead *ν*, the tunneling matrix element can be written as $\left(\Gamma_{i↑}^{\nu }=\Gamma_{i}^{\nu }(1+P_{\nu })\right)$ and $\left(\Gamma_{i↓}^{\nu }=\Gamma_{i}^{\nu }(1-P_{\nu })\right)$ with $\left(\Gamma_{i}^{\nu }=(\Gamma_{i↑}^{\nu }+\Gamma_{i↓}^{\nu })/2\right)$. $\left(\Gamma_{\gamma =1,2}=2\Pi \underset{k\sigma }{\sum }|T_{\gamma ,k}^{\left(i\right)}{|}^{2}N_{\gamma \sigma }\right)$, *N*_*γσ *_is the density of states when the superconductor is the normal state, and *ρ*_*γ*_(*ε*) is the modified BCS density of states $\left(\rho_{\gamma }(\varepsilon )\equiv \rho (\varepsilon )=\frac{\left|\varepsilon |\theta (|\varepsilon |-\Delta \right)}{\sqrt{\varepsilon^{2}-\Delta^{2}}}+\frac{\varepsilon \theta (\Delta -|\varepsilon |)}{i\sqrt{\Delta^{2}-\varepsilon^{2}}}\right)$. With the RSOI phase factor *φ*_R _=* φ*_R1_−*φ*_*R*2_, the spin-dependent phase factor is given by *ϕ*_*σ *_=* ϕ* + 2*σφ*_*R*_. We mainly take account of the case of the symmetric coupling between two superconducting electrodes and DQD, that is, Γ_*γ *_= Γ_s_. According to the fluctuation-dissipation theorem, the lesser self-energy can be given as $\left(\Sigma_{\nu }^{<}=F_{\nu }(\Sigma_{\nu }^{a}-\Sigma_{\nu }^{r})\right)$ and $\left(\Sigma_{S\gamma }^{<}=iF_{\gamma }\Gamma_{S\gamma }{\tilde{\rho }}_{\gamma }\right)$, where $\left({\tilde{\rho }}_{\gamma }\equiv \tilde{\rho }(\varepsilon )=(|\varepsilon |/\sqrt{\varepsilon^{2}-\Delta^{2}})\theta (|\varepsilon |-\Delta )\right)$. *F*_*ν*_ and *F*_*γ*_ are, respectively,

(19) $$\begin{array}{l} F_{\nu }=\left(\begin{array}{cccc} f_{\nu }(\varepsilon -eV_{\nu }) & 0 & 0 & 0\\ 0 & f_{\nu }(\varepsilon +eV_{\nu }) & 0 & 0\\ 0 & 0 & f_{\nu }(\varepsilon -eV_{\nu }) & 0\\ 0 & 0 & 0 & f_{\nu }(\varepsilon +eV_{\nu }) \end{array}\right), \end{array}$$

(20) $$\begin{array}{l} F_{\gamma }=\left(\begin{array}{cccc} f_{s}(\varepsilon ) & 0 & 0 & 0\\ 0 & f_{s}(\varepsilon ) & 0 & 0\\ 0 & 0 & f_{s}(\varepsilon ) & 0\\ 0 & 0 & 0 & f_{s}(\varepsilon ) \end{array}\right), \end{array}$$

in which *f*_*ν*_(*ε*−*eV*_*ν*_) =* f*_*ν*_, $\left(f_{\nu }(\varepsilon +eV_{\nu })={\bar{f}}_{\nu }\right)$, and *f*_*s*_(*ε*) are the Fermi distribution functions. By substituting Equations 15 to 20) into Equation 6, we can obtain the spin-related current as shown in Equations 9 and 10. The AR (CAR) coefficients ( $\left(T_{\sigma }^{\text{AR}}\right)$ and $\left(T_{\sigma }^{\text{CAR}}\right)$) and the probability of the quasiparticle tunneling ( $\left(T_{\sigma }^{\mathrm{LR}}\right)$ and $\left(T_{\sigma }^{\mathrm{QS}}\right)$) can be calculated as

(21) $$\begin{array}{l} T_{↑}^{\text{AR}}(\varepsilon )=\;\Gamma_{1↑}^{L}\Gamma_{1↓}^{L}|G_{12}^{r}(\varepsilon ){|}^{2}+\Gamma_{1↑}^{L}\Gamma_{2↓}^{L}|G_{14}^{r}(\varepsilon ){|}^{2}\\ \;\;\;+\Gamma_{2↑}^{L}\Gamma_{1↓}^{L}|G_{32}^{r}(\varepsilon ){|}^{2}+\Gamma_{2↑}^{L}\Gamma_{2↓}^{L}|G_{34}^{r}(\varepsilon ){|}^{2}\\ \;\;\;+2\alpha \Gamma_{1↓}^{L}\sqrt{\Gamma_{1↑}^{L}\Gamma_{2↑}^{L}}\text{Re}[e^{-i\phi /2}G_{12}^{r}(\varepsilon )G_{23}^{a}(\varepsilon )]\\ \;\;\;+2\alpha^{2}\sqrt{\Gamma_{1↑}^{L}\Gamma_{1↓}^{L}\Gamma_{2↑}^{L}\Gamma_{2↓}^{L}}\text{Re}[e^{-i\phi }G_{12}^{r}(\varepsilon )G_{43}^{a}(\varepsilon )]\\ \;\;\;+2\alpha^{2}\sqrt{\Gamma_{1↑}^{L}\Gamma_{1↓}^{L}\Gamma_{2↑}^{L}\Gamma_{2↓}^{L}}\text{Re}[G_{14}^{r}(\varepsilon )G_{23}^{a}(\varepsilon )]\\ \;\;\;+2\alpha \Gamma_{1↑}^{L}\sqrt{\Gamma_{1↓}^{L}\Gamma_{2↓}^{L}}\text{Re}[e^{-i\phi /2}G_{12}^{r}(\varepsilon )G_{41}^{a}(\varepsilon )]\\ \;\;\;+2\alpha \Gamma_{2↓}^{L}\sqrt{\Gamma_{1↑}^{L}\Gamma_{2↑}^{L}}\text{Re}[e^{-i\phi /2}G_{14}^{r}(\varepsilon )G_{43}^{a}(\varepsilon )]\\ \;\;\;+2\alpha \Gamma_{2↑}^{L}\sqrt{\Gamma_{1↓}^{L}\Gamma_{2↓}^{L}}\text{Re}[e^{-i\phi /2}G_{32}^{r}(\varepsilon )G_{43}^{a}(\varepsilon )], \end{array}$$

(22) $$\begin{array}{l} T_{↑}^{\mathrm{CAR}}(\varepsilon )=\Gamma_{1↑}^{L}\Gamma_{1↓}^{R}|G_{12}^{r}(\varepsilon ){|}^{2}+\Gamma_{1↑}^{L}\Gamma_{2↓}^{R}|G_{14}^{r}(\varepsilon ){|}^{2}\\ \;\;\;+\Gamma_{1↓}^{R}\Gamma_{2↑}^{L}|G_{32}^{r}(\varepsilon ){|}^{2}+\Gamma_{2↑}^{L}\Gamma_{2↓}^{R}|G_{34}^{r}(\varepsilon ){|}^{2}\\ \;\;\;+2\alpha \Gamma_{1↑}^{L}\sqrt{\Gamma_{1↓}^{R}\Gamma_{2↓}^{R}}\text{Re}[e^{-i\phi_{\sigma }/2}G_{12}^{r}(\varepsilon )G_{21}^{a}(\varepsilon )]\\ \;\;\;+2\alpha^{2}\sqrt{\Gamma_{1↑}^{L}\Gamma_{1↓}^{L}\Gamma_{2↑}^{R}\Gamma_{2↓}^{R}}\text{Re}[e^{i(\phi -\phi_{\sigma })/2}G_{34}^{r}(\varepsilon )G_{21}^{a}(\varepsilon )]\\ \;\;\;+2\alpha \Gamma_{2↓}^{R}\sqrt{\Gamma_{1↑}^{L}\Gamma_{2↑}^{L}}\text{Re}[e^{i\phi /2}G_{34}^{r}(\varepsilon )G_{41}^{a}(\varepsilon )]\\ \;\;\;+2\alpha^{2}\sqrt{\Gamma_{1↑}^{L}\Gamma_{1↓}^{L}\Gamma_{1↓}^{R}\Gamma_{2↓}^{R}}\text{Re}[e^{i(\phi +\phi_{\sigma })}G_{32}^{r}(\varepsilon )G_{41}^{a}(\varepsilon )]\\ \;\;\;+2\alpha \Gamma_{1↓}^{R}\sqrt{\Gamma_{1↑}^{L}\Gamma_{2↑}^{L}}\text{Re}[e^{-i\phi /2}G_{12}^{r}(\varepsilon )G_{23}^{a}(\varepsilon )]\\ \;\;\;+2\alpha \Gamma_{2↑}^{L}\sqrt{\Gamma_{1↓}^{R}\Gamma_{2↓}^{R}}\text{Re}[e^{-i\phi_{\sigma }/2}G_{34}^{r}(\varepsilon )G_{23}^{a}(\varepsilon )], \end{array}$$

(23) $$\begin{array}{l} T_{↑}^{\mathrm{LR}}(\varepsilon )=\Gamma_{1↑}^{L}\Gamma_{1↓}^{R}|G_{11}^{r}(\varepsilon ){|}^{2}+\Gamma_{1↑}^{L}\Gamma_{2↑}^{R}|G_{13}^{r}(\varepsilon ){|}^{2}\\ \;\;\;+\Gamma_{1↑}^{R}\Gamma_{2↑}^{L}|G_{31}^{r}(\varepsilon ){|}^{2}+\Gamma_{2↑}^{L}\Gamma_{2↑}^{R}|G_{33}^{r}(\varepsilon ){|}^{2}\\ \;\;\;+2\alpha \Gamma_{1↑}^{L}\sqrt{\Gamma_{1↑}^{R}\Gamma_{2↑}^{R}}\text{Re}[e^{i\phi_{\sigma }/2}G_{13}^{r}(\varepsilon )G_{11}^{a}(\varepsilon )]\\ \;\;\;+2\alpha^{2}\sqrt{\Gamma_{1↑}^{L}\Gamma_{2↑}^{L}\Gamma_{1↑}^{R}\Gamma_{2↑}^{R}}\text{Re}[e^{i(\phi_{\sigma }+\phi )/2}G_{33}^{r}(\varepsilon )G_{11}^{a}(\varepsilon )]\\ \;\;\;+2\alpha^{2}\sqrt{\Gamma_{1↑}^{L}\Gamma_{2↑}^{L}\Gamma_{1↑}^{R}\Gamma_{2↑}^{R}}\text{Re}[e^{i(\phi_{\sigma }-\phi )/2}G_{13}^{r}(\varepsilon )G_{13}^{a}(\varepsilon )]\\ \;\;\;+2\alpha \Gamma_{2↑}^{R}\sqrt{\Gamma_{1↑}^{L}\Gamma_{2↑}^{L}}\text{Re}[e^{i\phi /2}G_{33}^{r}(\varepsilon )G_{31}^{a}(\varepsilon )]\\ \;\;\;+2\alpha \Gamma_{2↑}^{L}\sqrt{\Gamma_{1↑}^{R}\Gamma_{2↑}^{R}}\text{Re}[e^{i\phi_{\sigma }/2}G_{33}^{r}(\varepsilon )G_{13}^{a}(\varepsilon )]\\ \;\;\;+2\alpha \Gamma_{1↑}^{R}\sqrt{\Gamma_{1↑}^{L}\Gamma_{2↑}^{L}}\text{Re}[e^{i\phi /2}G_{31}^{r}(\varepsilon )G_{11}^{a}(\varepsilon )], \end{array}$$

(24) $$\begin{array}{l} T_{↑}^{\mathrm{QS}}(\varepsilon )=\Gamma_{s}\tilde{\rho }\{\Gamma_{1↑}^{L}[|G_{11}^{r}(\varepsilon ){|}^{2}+|G_{12}^{r}(\varepsilon ){|}^{2}+|G_{13}^{r}(\varepsilon ){|}^{2}\\ \;\;\;+|G_{14}^{r}(\varepsilon ){|}^{2}]+\Gamma_{2↑}^{L}[|G_{31}^{r}(\varepsilon ){|}^{2}+|G_{32}^{r}(\varepsilon ){|}^{2}\\ \;\;\;+|G_{33}^{r}(\varepsilon ){|}^{2}+|G_{34}^{r}(\varepsilon ){|}^{2}]\\ \;\;\;+2\alpha \sqrt{\Gamma_{1↑}^{L}\Gamma_{2↑}^{L}}\text{Re}[e^{i\phi /2}G_{32}^{r}(\varepsilon )G_{21}^{a}(\varepsilon )]\\ \;\;\;+2\alpha \sqrt{\Gamma_{1↑}^{L}\Gamma_{2↑}^{L}}\text{Re}[e^{i\phi /2}G_{33}^{r}(\varepsilon )G_{31}^{a}(\varepsilon )]\\ \;\;\;+2\alpha \sqrt{\Gamma_{1↑}^{L}\Gamma_{2↑}^{L}}\text{Re}[e^{i\phi /2}G_{34}^{r}(\varepsilon )G_{41}^{a}(\varepsilon )]\\ \;\;\;-2\Gamma_{1↑}^{L}\frac{\Delta }{\varepsilon }\text{Re}[G_{12}^{r}(\varepsilon )G_{11}^{a}(\varepsilon )+G_{14}^{r}(\varepsilon )G_{31}^{a}(\varepsilon )]\\ \;\;\;-2\Gamma_{2↑}^{L}\frac{\Delta }{\varepsilon }\text{Re}[G_{32}^{r}(\varepsilon )G_{13}^{a}(\varepsilon )+G_{33}^{r}(\varepsilon )G_{43}^{a}(\varepsilon )]\\ \;\;\;-2\alpha \sqrt{\Gamma_{1↑}^{L}\Gamma_{2↑}^{L}}\text{Re}[e^{i\phi /2}G_{31}^{r}(\varepsilon )G_{21}^{a}(\varepsilon )\\ \;\;\;+e^{i\phi /2}G_{34}^{r}(\varepsilon )G_{31}^{a}(\varepsilon )]\\ \;\;\;-2\alpha \sqrt{\Gamma_{1↑}^{L}\Gamma_{2↑}^{L}}\text{Re}[e^{i\phi /2}G_{32}^{r}(\varepsilon )G_{11}^{a}(\varepsilon )\\ \;\;\;+e^{i\phi /2}G_{33}^{r}(\varepsilon )G_{41}^{a}(\varepsilon )]\}, \end{array}$$

(25) $$\begin{array}{l} T_{↓}^{\text{AR}}(\varepsilon )=\Gamma_{1↑}^{L}\Gamma_{1↓}^{L}|G_{21}^{r}(\varepsilon ){|}^{2}+\Gamma_{2↑}^{L}\Gamma_{1↓}^{L}|G_{23}^{r}(\varepsilon ){|}^{2}\\ \;\;\;+\Gamma_{1↑}^{L}\Gamma_{2↓}^{L}|G_{41}^{r}(\varepsilon ){|}^{2}+\Gamma_{2↑}^{L}\Gamma_{2↓}^{L}|G_{43}^{r}(\varepsilon ){|}^{2}\\ \;\;\;+2\alpha \Gamma_{1↓}^{L}\sqrt{\Gamma_{1↑}^{L}\Gamma_{2↑}^{L}}\text{Re}[e^{-i\phi /2}G_{23}^{r}(\varepsilon )G_{12}^{a}(\varepsilon )]\\ \;\;\;+2\alpha^{2}\sqrt{\Gamma_{1↑}^{L}\Gamma_{1↓}^{L}\Gamma_{2↑}^{L}\Gamma_{2↓}^{L}}\text{Re}[e^{-i\phi }G_{43}^{r}(\varepsilon )G_{12}^{a}(\varepsilon )]\\ \;\;\;+2\alpha^{2}\sqrt{\Gamma_{1↑}^{L}\Gamma_{1↓}^{L}\Gamma_{2↑}^{L}\Gamma_{2↓}^{L}}\text{Re}[G_{41}^{r}(\varepsilon )G_{32}^{a}(\varepsilon )]\\ \;\;\;+2\alpha \Gamma_{1↑}^{L}\sqrt{\Gamma_{1↓}^{L}\Gamma_{2↓}^{L}}\text{Re}[e^{-i\phi /2}G_{41}^{r}(\varepsilon )G_{12}^{a}(\varepsilon )]\\ \;\;\;+2\alpha \Gamma_{2↓}^{L}\sqrt{\Gamma_{1↑}^{L}\Gamma_{2↑}^{L}}\text{Re}[e^{-i\phi /2}G_{43}^{r}(\varepsilon )G_{14}^{a}(\varepsilon )]\\ \;\;\;+2\alpha \Gamma_{2↑}^{L}\sqrt{\Gamma_{1↓}^{L}\Gamma_{2↓}^{L}}\text{Re}[e^{-i\phi /2}G_{32}^{r}(\varepsilon )G_{43}^{a}(\varepsilon )],\; \end{array}$$

(26) $$\begin{array}{l} T_{↓}^{\mathrm{CAR}}(\varepsilon )=\Gamma_{1↓}^{L}\Gamma_{1↑}^{R}|G_{21}^{r}(\varepsilon ){|}^{2}+\Gamma_{1↓}^{L}\Gamma_{2↑}^{R}|G_{23}^{r}(\varepsilon ){|}^{2}\\ \;\;\;+\Gamma_{1↑}^{R}\Gamma_{2↓}^{L}|G_{41}^{r}(\varepsilon ){|}^{2}+\Gamma_{2↓}^{L}\Gamma_{2↑}^{R}|G_{43}^{r}(\varepsilon ){|}^{2}\\ \;\;\;+2\alpha \Gamma_{1↓}^{L}\sqrt{\Gamma_{1↑}^{R}\Gamma_{2↑}^{R}}\text{Re}[e^{i\phi_{\sigma }/2}G_{23}^{r}(\varepsilon )G_{12}^{a}(\varepsilon )]\\ \;\;\;+2\alpha^{2}\sqrt{\Gamma_{1↓}^{L}\Gamma_{2↓}^{L}\Gamma_{1↑}^{R}\Gamma_{2↑}^{R}}\text{Re}[e^{i(\phi_{\sigma }-\phi )/2}G_{43}^{r}(\varepsilon )G_{12}^{a}(\varepsilon )]\\ \;\;\;+2\alpha^{2}\sqrt{\Gamma_{1↓}^{L}\Gamma_{2↓}^{L}\Gamma_{1↑}^{R}\Gamma_{2↑}^{R}}\text{Re}[e^{i(\phi_{\sigma }+\phi )/2}G_{23}^{r}(\varepsilon )G_{14}^{a}(\varepsilon )]\\ \;\;\;+2\alpha \Gamma_{2↑}^{R}\sqrt{\Gamma_{1↓}^{L}\Gamma_{2↓}^{L}}\text{Re}[e^{i\phi /2}G_{23}^{r}(\varepsilon )G_{34}^{a}(\varepsilon )]\\ \;\;\;+2\alpha \Gamma_{1↑}^{R}\sqrt{\Gamma_{1↓}^{L}\Gamma_{2↓}^{L}}\text{Re}[e^{i\phi /2}G_{21}^{r}(\varepsilon )G_{14}^{a}(\varepsilon )]\\ \;\;\;+2\alpha \Gamma_{2↓}^{L}\sqrt{\Gamma_{1↑}^{R}\Gamma_{2↑}^{R}}\text{Re}[e^{i\phi_{\sigma }/2}G_{43}^{r}(\varepsilon )G_{14}^{a}(\varepsilon )]\; \end{array}$$

(27) $$\begin{array}{l} T_{↓}^{\mathrm{LR}}(\varepsilon )=\Gamma_{1↓}^{L}\Gamma_{1↓}^{R}|G_{22}^{r}(\varepsilon ){|}^{2}+\Gamma_{1↓}^{L}\Gamma_{2↓}^{R}|G_{24}^{r}(\varepsilon ){|}^{2}\\ \;\;\;+\Gamma_{1↓}^{R}\Gamma_{2↓}^{L}|G_{42}^{r}(\varepsilon ){|}^{2}+\Gamma_{2↓}^{L}\Gamma_{2↓}^{R}|G_{44}^{r}(\varepsilon ){|}^{2}\\ \;\;\;+2\alpha \Gamma_{1↓}^{R}\sqrt{\Gamma_{1↓}^{L}\Gamma_{2↓}^{L}}\text{Re}[e^{i\phi_{\sigma }/2}G_{22}^{r}(\varepsilon )G_{24}^{a}(\varepsilon )]\\ \;\;\;+2\alpha^{2}\sqrt{\Gamma_{1↓}^{L}\Gamma_{2↓}^{L}\Gamma_{1↓}^{R}\Gamma_{2↓}^{R}}\text{Re}[e^{-i(\phi_{\sigma }+\phi )/2}G_{44}^{r}(\varepsilon )G_{22}^{a}(\varepsilon )]\\ \;\;\;+2\alpha^{2}\sqrt{\Gamma_{1↓}^{L}\Gamma_{2↓}^{L}\Gamma_{1↓}^{R}\Gamma_{2↓}^{R}}\text{Re}[e^{i(\phi_{\sigma }-\phi )/2}G_{24}^{r}(\varepsilon )G_{24}^{a}(\varepsilon )]\\ \;\;\;+2\alpha \Gamma_{2↓}^{R}\sqrt{\Gamma_{1↓}^{L}\Gamma_{2↓}^{L}}\text{Re}[e^{i\phi /2}G_{24}^{r}(\varepsilon )G_{44}^{a}(\varepsilon )]\\ \;\;\;+2\alpha \Gamma_{2↓}^{L}\sqrt{\Gamma_{1↓}^{R}\Gamma_{2↓}^{R}}\text{Re}[e^{-i\phi_{\sigma }/2}G_{44}^{r}(\varepsilon )G_{24}^{a}(\varepsilon )]\\ \;\;\;+2\alpha \Gamma_{1↓}^{L}\sqrt{\Gamma_{1↑}^{R}\Gamma_{2↑}^{R}}\text{Re}[e^{-i\phi_{\sigma }/2}G_{24}^{r}(\varepsilon )G_{22}^{a}(\varepsilon )],\; \end{array}$$

(28) $$\begin{array}{l} T_{↓}^{\mathrm{QS}}(\varepsilon )=\Gamma_{s}\tilde{\rho }\{\Gamma_{1↓}^{L}[|G_{21}^{r}(\varepsilon ){|}^{2}+|G_{22}^{r}(\varepsilon ){|}^{2}+|G_{23}^{r}(\varepsilon ){|}^{2}+|G_{24}^{r}(\varepsilon ){|}^{2}]\\ \;\;\;+\Gamma_{2↓}^{L}[|G_{41}^{r}(\varepsilon ){|}^{2}+|G_{42}^{r}(\varepsilon ){|}^{2}+|G_{43}^{r}(\varepsilon ){|}^{2}+|G_{44}^{r}(\varepsilon ){|}^{2}]\\ \;\;\;+2\alpha \sqrt{\Gamma_{1↓}^{L}\Gamma_{2↓}^{L}}\text{Re}[e^{-i\phi /2}G_{41}^{r}(\varepsilon )G_{12}^{a}(\varepsilon )]\\ \;\;\;+2\alpha \sqrt{\Gamma_{1↓}^{L}\Gamma_{2↓}^{L}}\text{Re}[e^{-i\phi /2}G_{42}^{r}(\varepsilon )G_{22}^{a}(\varepsilon )]\\ \;\;\;+2\alpha \sqrt{\Gamma_{1↓}^{L}\Gamma_{2↓}^{L}}\text{Re}[e^{-i\phi /2}G_{43}^{r}(\varepsilon )G_{32}^{a}(\varepsilon )]\\ \;\;\;+2\alpha \sqrt{\Gamma_{1↓}^{L}\Gamma_{2↓}^{L}}\text{Re}[e^{-i\phi /2}G_{44}^{r}(\varepsilon )G_{42}^{a}(\varepsilon )]\\ \;\;\;-2\Gamma_{1↓}^{L}\frac{\Delta }{\varepsilon }\text{Re}[G_{22}^{r}(\varepsilon )G_{12}^{a}(\varepsilon )+G_{24}^{r}(\varepsilon )G_{32}^{a}(\varepsilon )]\\ \;\;\;-2\Gamma_{2↓}^{L}\frac{\Delta }{\varepsilon }\text{Re}[G_{42}^{r}(\varepsilon )G_{14}^{a}(\varepsilon )+G_{44}^{r}(\varepsilon )G_{34}^{a}(\varepsilon )]\\ \;\;\;-2\alpha \sqrt{\Gamma_{1↓}^{L}\Gamma_{2↓}^{L}}\text{Re}[e^{-i\phi /2}G_{42}^{r}(\varepsilon )G_{12}^{a}(\varepsilon )\\ \;\;\;+e^{-i\phi /2}G_{44}^{r}(\varepsilon )G_{32}^{a}(\varepsilon )]\\ \;\;\;-2\alpha \sqrt{\Gamma_{1↓}^{L}\Gamma_{2↓}^{L}}\text{Re}[e^{-i\phi /2}G_{41}^{r}(\varepsilon )G_{22}^{a}(\varepsilon )\\ \;\;\;+e^{-i\phi /2}G_{43}^{r}(\varepsilon )G_{42}^{a}(\varepsilon )]\}.\; \end{array}$$

Thus, we can investigate the quantum transport through our model system based on the above-mentioned equations.

## Competing interests

The authors declare that they have no competing interests.

## Authors’ contributions

LB established the physical model and the theoretical formalism. RZ and CLD carried out the numerical calculations and the establishment of the figures. LB performed the physical analysis and revised the manuscript. All the authors read and approved the final manuscript.

## References

[B1] Reimann SM Manninen M Electronic structure of quantum dots Rev Mod Phys 2002 74 1283 10.1103/RevModPhys.74.1283

[B2] Wang ZM Self-Assembled Quantum Dots 2008 New York: Springer

[B3] Hanson R Kouwenhoven LP Petta JR Tarucha S Vandersypen LMK Spins in few-electron quantum dots Rev Mod Phys 2007 79 1212

[B4] Andergassen S Meden V Schoeller H Splettstoesse J Wegewijs MR Charge transport through single molecules, quantum dots and quantum wires Nanotechnology 2010 21 272001 10.1088/0957-4484/21/27/272001 20571187

[B5] Aleiner IL Brouwer PW Glazman LI Quantum effects in Coulomb blockade Phys Rep 2002 358 309 10.1016/S0370-1573(01)00063-1

[B6] Dubi Y Di Ventra M Heat flow and thermoelectricity in atomic and molecular junctions Rev Mod Phys 2011 83 131 10.1103/RevModPhys.83.131

[B7] Lu HZ Lü R Zhu BF Tunable Fano effect in parallel-coupled double quantum dot system Phys Rev B 2005 71 235320

[B8] Trocha P Barnas J Quantum interference and Coulomb correlation effects in spin-polarized transport through two coupled quantum dots Phys Rev B 2007 76 165432

[B9] Kubala B Konig BK Flux-dependent level attraction in double-dot Aharonov-Bohm interferometers Phys Rev B 2002 65 245301

[B10] Chi F Yuan XQ Zheng J Double Rashba quantum dots ring as a spin filter Nanoscale Res Lett 2008 3 343 10.1007/s11671-008-9163-z

[B11] Liu YS Chen H Yang XF Transport properties of an Aharonov-Bohm ring with strong interdot Coulomb interaction J Phys Condens Matter 2007 19 246201 10.1088/0953-8984/19/24/246201 21694045

[B12] Zitko R Mravlje J Haule K Ground state of the parallel double quantum dot system Phys Rev Lett 2012 108 066602 22401099 10.1103/PhysRevLett.108.066602

[B13] Fang TF Luo HG Tuning the Kondo and Fano effects in double quantum dots Phys Rev B 2010 81 113402

[B14] Krause T Schaller G Brandes T Incomplete current fluctuation theorems for a four-terminal model Phys Rev B 2011 84 195113

[B15] Loss D Sukhorukov EV Probing entanglement and nonlocality of electrons in a double-dot via transport and noise Phys Rev Lett 2000 84 1035 10.1103/PhysRevLett.84.1035 11017434

[B16] Smirnov AY Horing NJM Mourokh LG Aharonov-Bohm phase effects and inelastic scattering in transport through a parallel tunnel-coupled symmetric double-dot device Appl Phys Lett 2578 77 2000

[B17] Sukhorukov EV Burkard G Loss D Noise of a quantum dot system in the cotunneling regime Phys Rev B 2001 63 125315

[B18] Mourokh LG Horing NJM Smirnov AY Electron transport through a parallel double-dot system in the presence of Aharonov-Bohm flux and phonon scattering Phys Rev B 2002 66 085332

[B19] Holleitner AW Decker CR Qin H Eberl K Blick RH Coherent coupling of two quantum dots embedded in an Aharonov-Bohm interferometer Phys Rev Lett 2001 87 256802 11736594 10.1103/PhysRevLett.87.256802

[B20] Holleitner AW Blick RH Huttel AK Eberl K Kotthaus JP Probing and controlling the bonds of an artificial molecule Science 2002 297 70 10.1126/science.1071215 12098692

[B21] Kubo T Tokura Y Hatano T Tarucha S Electron transport through Aharonov-Bohm interferometer with laterally coupled double quantum dots Phys Rev B 2006 74 205310

[B22] Gurvitz SA Quantum interference in resonant tunneling single spin measurements IEEE Trans Nanotechol 2005 4 45 10.1109/TNANO.2004.840151

[B23] Kubo T Tokura Y Hatano T Tarucha S Exotic pseudospin Kondo effect in laterally coupled double quantum dots Phys Rev B 2008 77 041305(R)

[B24] Kubo T Tokura Y Hatano T Tarucha S Kondo effects and shot noise enhancement in a laterally coupled double quantum dot Phys Rev B 2011 83 115310

[B25] Trocha P The role of the indirect tunneling processes and asymmetry in couplings in orbital Kondo transport through double quantum dots J Phys Condens Matter 2012 24 055303 10.1088/0953-8984/24/5/055303 22248545

[B26] Sun QF Xie XC Bias-controllable intrinsic spin polarization in a quantum dot: proposed scheme based on spin-orbit interaction Phys Rev B 2006 73 235301

[B27] Sun QF Wang J Guo H Quantum transport theory for nanostructures with Rashba spin-orbital interaction Phys Rev B 2005 71 165310

[B28] Tserkovnyak Y Akhanjee S Spin-selective localization due to intrinsic spin-orbit coupling Phys Rev B 2009 79 085114

[B29] Wu MW Jiang JH Weng MQ Spin dynamics in semiconductors Phys Rep 2010 493 61 10.1016/j.physrep.2010.04.002

[B30] Stepanenko D Rudner M Halperin BI Loss D Singlet-triplet splitting in double quantum dots due to spin-orbit and hyperfine interactions Phys Rev B 2012 85 075416

[B31] Sun QF Wang J Lin TH Resonant Andreev reflection in a normal-metal-quantum dot-supercoductor system Phys Rev B 1999 59 3831 10.1103/PhysRevB.59.3831

[B32] Koerting V Andersen BM Flensberg K Paaske J Nonequilibrium transport via spin-induced subgap states in superconductor/quantum dot/normal metal cotunnel junctions Phys Rev B 2010 82 245108

[B33] Baranski J Domanski T Fano-type interference in quantum dots coupled between metallic and superconducting leads Phys Rev B 2011 84 195424

[B34] Xing YX Wang J Universal conductance fluctuations in mesoscopic systems with superconducting leads: beyond the Andreev approximation Phys Rev B 2010 82 245406

[B35] Whitney RS Jacquod P Controlling the sign of magnetoconductance in Andreev quantum dots Phys Rev Lett 2009 103 247002 20366223 10.1103/PhysRevLett.103.247002

[B36] Skadsem HJ Brataas A Martinek J Tserkovnyak Y Ferromagnetic resonance and voltage-induced transport in normal metal-ferromagnet-superconductor trilayers Phys Rev B 2011 84 104420

[B37] Golubov AA Tanaka Y Mazin II Dolgov OV Brinkman Andreev spectra and subgap bound states in multiband superconductors Phys Rev Lett 2009 103 077003 10.1103/PhysRevLett.103.077003 19792677

[B38] Annunziata G Cuoco M Noce C Sudbo A Linder J Spin-sensitive long-range proximity effect in ferromagnet/spin-triplet-superconductor bilayers Phys Rev B 2011 83 060508(R)

[B39] Morten JP Brataas A Belzig W Circuit theory of crossed Andreev reflection Phys Rev B 2006 74 214510

[B40] Golubev DS Zaikin AD Non-local Andreev reflection in superconducting quantum dots Phys Rev B 2007 76 184510

[B41] Sothmann B Futterer D Governale M Konig J Probing the exchange field of a quantum-dot spin valve by a superconducting lead Phys Rev B 2010 82 094514

[B42] Futterer D Governale M Pala MG Konig J Nonlocal Andreev transport through an interacting quantum dot Phys Rev B 2009 79 054505

[B43] Brauer J Hubler F Smetanin M Beckmann D Lohneysen HV Nonlocal transport in normal-metal/superconductor hybrid structures: role of interference and interaction Phys Rev B 2010 81 024515

[B44] Hofstetter L Csonka S Nygardand C Schonenberger S Cooper pair splitter realized in a two-quantum-dot Y-junction Nature 2009 461 960 10.1038/nature08432 19829377

[B45] Herrmann LG Portier F Roche P Yeyati AL Kontos T Strunk C Carbon nanotubes as Cooper-pair beam splitters Phys Rev Lett 2010 104 026801 20366615 10.1103/PhysRevLett.104.026801

[B46] Nitta J Akazaki T Takayanagi H Enoki T Gate control of spin-orbit interaction in an inverted In0.53Ga0.47As/In0.52Al0.48As heterostructure Phys Rev Lett 1997 78 1335 10.1103/PhysRevLett.78.1335

[B47] Matsuyama T Kursten R Meissner C Merkt U Rashba spin splitting in inversion layers on p-type bulk InAs Phys Rev B 2000 61 15588 10.1103/PhysRevB.61.15588

[B48] Jauho AP Haug H Quantum Kinetics in Transport and Optics of Semiconductors 2008 Berlin: Springer

[B49] Jauho AP Wingreen NS Meir Y Time-dependent transport in interacting and noninteracting resonant-tunneling systems Phys Rev B 1994 50 5528 10.1103/PhysRevB.50.5528 9976896

